# Clinical Outcomes and Genomic Alterations in Gleason Score 10 Prostate Cancer

**DOI:** 10.3390/cancers17071055

**Published:** 2025-03-21

**Authors:** Luke W. Chen, Yetkin Tuac, Sophia Li, Jonathan E. Leeman, Martin T. King, Peter F. Orio, Paul L. Nguyen, Anthony V. D’Amico, Cagdas Aktan, Mutlay Sayan

**Affiliations:** 1 Department of Radiation Oncology, Brigham and Women’s Hospital and Dana Farber Cancer Institute, Harvard Medical School, Boston, MA 02115, USA; 2 Department of Statistics, Ankara University, 06100 Ankara, Türkiye; 3 Department of Medical Biology, Faculty of Medicine, Bandirma Onyedi Eylul University, 10250 Balikesir, Türkiye

**Keywords:** prostate cancer, Gleason score, disease-free survival, gene expression profiling, biomarkers

## Abstract

**Background**: Gleason score (GS) 10 prostate cancer (PC) is a highly aggressive localized disease. Despite advances in treating high-risk PC, the clinical outcomes and molecular underpinnings of GS 10 remain unclear. This study aimed to determine whether GS 10 PC has distinct clinical outcomes from other “high-risk” cancers (i.e., Gleason 8–9) and identify genomic alterations driving its aggressive phenotype. **Methods**: A retrospective review of The Cancer Genome Atlas database identified patients with GS 8–10 PC who underwent radical prostatectomy. Clinical factors were compared between GS 10 and GS 8–9 cohorts. Time to biochemical recurrence (BCR) was analyzed using Kaplan–Meier and Cox regression. RNA sequencing identified differentially expressed genes, and protein–protein interaction networks identified hub genes. **Results**: Of 192 patients, 13 (6.8%) had GS 10 PC. After median follow-up of 37.87 months, GS 10 status was associated with significantly lower time to BCR (AHR, 2.67; 95% CI, 1.18–6.02; *p* = 0.018) compared to GS 8–9. Multiple genes (e.g., *RAD54L*, *FAAH*, *AATK*, *MAST2*) showed higher alteration frequencies, and high expression of *RAD54L*, *MAST2*, and *CCHCR1* correlated with shorter disease-free survival. Six overlapping hub genes (*CD8A*, *CDC20*, *E2F1*, *IL10*, *TNF*, *VCAM1*) were overexpressed in GS 10 tumors, reflecting key pathways in tumor progression. **Conclusions**: GS 10 PC confers inferior time to BCR and displays a distinct genomic landscape compared to GS 8–9 disease, highlighting the need for biomarker-driven therapeutic strategies. Further studies are needed to validate these genomic targets and improve management for this very high-risk population.

## Simple Summary

Prostate cancer with a Gleason score of 10 is rare yet highly aggressive and may not respond well to standard treatments. This study examined patients who had surgery for prostate cancer and found that those with a Gleason score of 10 had a shorter time to biochemical recurrence compared to those with lower Gleason scores. The research also identified specific genetic changes that could explain the aggressive nature of this form of prostate cancer. Pinpointing these differences is an important step toward developing tests for earlier detection and designing more personalized therapies, thereby helping these patients achieve better long-term outcomes.

## 1. Introduction

The Gleason grading system remains one of the most important prognostic factors in prostate cancer (PC), with Gleason score (GS) 10 representing the most aggressive form of clinically localized disease [1]. GS 10 PC, composed entirely of poorly differentiated pattern 5 tumor, accounts for a small percentage of all newly diagnosed PCs [2,3]. While significant progress has been made in improving outcomes for high-risk PC in general through multimodal treatment approaches [4,5,6], the specific subset of patients with GS 10 PC has been understudied. The rarity of GS 10 PC has resulted in its inclusion with GS 9 disease in most prior studies, potentially obscuring the unique clinical features of this very high-risk group.

A previous study by Kishan et al. showed that within 5 years, 24% of the cohort of GS 9–10 patients who underwent radical prostatectomy (RP) developed distant metastasis and 12% died [7]. It is possible that patients with GS 10 PC may have even worse outcomes than the broader GS 8–10 group which defines high-risk PC. This may be due to a high likelihood of micrometastatic disease beyond the pelvis at diagnosis, which is not adequately addressed by current treatment paradigms focused on locoregional control. Additionally, GS 10 tumors may harbor distinct molecular alterations driving treatment resistance. Identifying such alterations would be an important step towards a greater understanding of the aggressive biology of GS 10 PC and designing more targeted, effective treatments.

In this study, we first compare patients who underwent RP for GS 10 PC to those with GS 8–9 disease, aiming to clarify whether GS 10 status confers distinct clinical outcomes. We then examine genomic data and protein–protein interactions to identify key genes that might underlie the observed variations in disease-free survival, thereby providing novel insights into potential therapeutic targets for this very high-risk patient population.

## 2. Materials and Methods

### 2.1. Study Population

Patient records were retrospectively obtained from The Cancer Genome Atlas (TCGA) prostate adenocarcinoma database [8]. Patients included in this study met the following criteria: (1) a diagnosis of prostate adenocarcinoma, (2) treatment with radical prostatectomy, and (3) a Gleason score of 8 to 10. Patients who did not meet these criteria were excluded from the analysis. The follow-up time was defined from the date of RP to the last documented follow-up or death.

### 2.2. Comparison of Clinical Factors Stratified by Gleason Score

A predefined statistical analysis plan was established and followed throughout the study. Patient clinical and treatment characteristics were identified using descriptive statistics, stratifying patients based on their Gleason score (GS 8–9 vs. 10). Categorical covariates were compared using Pearson’s chi-squared test, and continuous covariates were analyzed using the Wilcoxon two-sample [9]. The reverse Kaplan–Meier method was used to compare follow-up time distributions; log-rank *p*-value was used to analyze significance [10].

### 2.3. Covariate-Adjusted Hazard Ratios and Estimates of Time to Biochemical Recurrence

The primary endpoint of this study was time to biochemical recurrence (BCR), measured from RP to the first detected occurrence of BCR. To determine whether a significant association existed between GS 10 status and time to BCR, univariable and multivariable Cox regression were used. The model included covariates such as age at diagnosis, pre-RP prostate-specific antigen (PSA), pathologic tumor stage, margin status, and nodal status. The date of RP was set as Time 0. The Kaplan–Meier method was used to generate covariate-adjusted estimates of time to BCR following RP, stratified by GS 8–9 vs. 10 [11]. The log-rank test was used to generate *p*-values for the adjusted Kaplan–Meier curves, with statistical significance set at *p* < 0.05. All statistical analyses were performed with R (version 4.2.3).

### 2.4. Genomic Alterations and Gene Expression Analysis

In order to analyze genomic alterations, mutation and copy number alteration data were extracted from the database. Fresh-frozen and formalin-fixed paraffin-embedded tissues were analyzed through DNA extraction and next-generation sequencing to generate the data in the database. Differentially altered genes (DAGs) were identified in patients based on significantly higher alteration event frequencies in GS 10 patients compared to GS 8–9 patients, using *p* value threshold of <0.05.

The mRNA expression levels on prostate cancer tissue were analyzed using gene expression profiling data obtained from the database. RNA sequencing (RNASeq V2) data, processed and normalized through RNA-Seq by Expectation–Maximization (RSEM), were used to identify differentially expressed genes (DEGs) [8]. DEGs were identified in patients with GS 8–9 and GS 10 based on a *p*-value threshold of <0.05 and log2 fold change (log2FC) < −0.58 or >0.58 [12].

### 2.5. Construction of Protein–Protein Interaction Network and Identification of Hub Genes

The protein–protein interaction (PPI) network was constructed using the Search Tool for the Retrieval of Interacting Genes v12.0 (https://string-db.org/, accessed on 29 September 2024), and the PPI network was visualized using Cytoscape (v3.10.2) [13,14]. The molecular complex detection (MCODE) plug-in (v2.0.3) in Cytoscape was used to identify significant genes, using a K-score of 2, a degree cutoff of 2, a node cutoff of 0.2, and a maximum depth of 100 as the parameters [15]. The Cytohubba plug-in (v0.1) was then applied to identify the most intersected key genes and modules [16]. Hub genes—highly interconnected genes within the PPI network—were identified based on their significance in Cytohubba algorithms of degree, closeness and betweenness.

### 2.6. The Prognostic Value of the Altered and Hub Genes

The log-rank test on GEPIA (http://gepia.cancer-pku.cn/, accessed on 10 October 2024) was used to analyze disease-free survival (DFS) based on the DAGs and hub genes. The Cox proportional hazard ratio (HR) and 95% confidence intervals (CIs) were reported for the survival plots.

### 2.7. Functional Enrichment Analysis

Functional enrichment analysis was conducted using gene ontology (GO) analysis to determine the biological significance of the differentially expressed hub genes. Gene functionalities were classified into three categories: biological processes, molecular functions, and cellular components. Annotation and pathway enrichment analysis of hub genes were conducted using the comprehensive gProfiler web tool (https://biit.cs.ut.ee/gprofiler/gost, accessed on 10 March 2025), utilizing the KEGG pathway database to facilitate this analysis [17].

## 3. Results

### 3.1. Comparison of the Distribution of Clinical Factors Stratified by Gleason Score

Among the 192 patients analyzed, 13 (6.77%) had a GS of 10. The patient characteristics, stratified by the GS, are summarized in Table 1. Compared to those with GS 8–9, patients with GS 10 PC had a longer median follow-up [43.40 months (IQR: 19.30, 70.23) versus 37.87 months (IQR: 16.53, 64.37), *p* = 0.002], higher pre-RP PSA levels [14.20 ng/mL (IQR: 8.20, 64.10) versus 8.90 ng/mL (IQR: 5.35, 14.80), *p* = 0.038], and a higher rate of positive prostatectomy margins (100% versus 50%, *p* < 0.001).

### 3.2. Covariate-Adjusted Hazard Ratios and Estimates of Time to Biochemical Recurrence

After a median follow-up of 37.87 months (IQR: 17.07, 64.37), BCR occurred in 64 men (33.33%). As shown in Table 2, GS 10 was associated with a significantly lower time to BCR (AHR, 2.67; 95% CI, 1.18 to 6.02; *p* = 0.018) after adjusting for known covariates.

**Figure 1:**
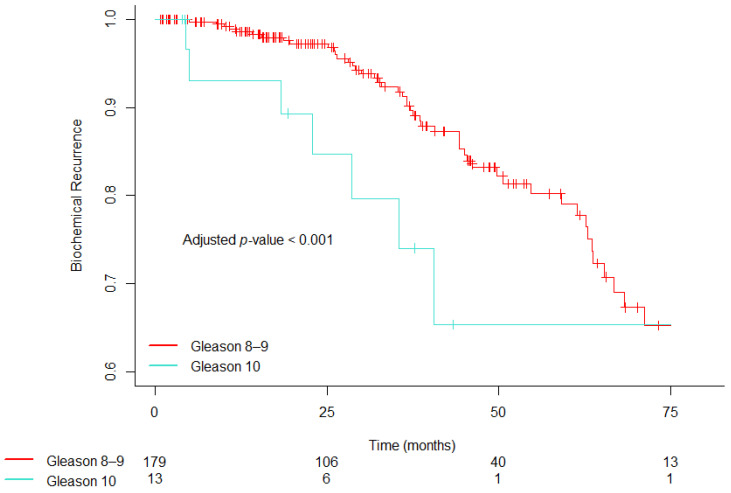
Covariate-adjusted estimates of time to biochemical recurrence stratified by Gleason score.

Figure 1 displays a plot comparing the estimated time to BCR for the two Gleason Score patient groups (GS 8–9 and GS 10) after adjusting for covariates, utilizing the Kaplan–Meier method. Specifically, Figure 1 illustrates that patients with GS 10 had significantly lower adjusted time to BCR estimates compared to those with GS 8–9 (*p* < 0.001). The adjusted 5-year freedom from BCR was 65.40% (95% CI, 35.40% to 100.0%) for patients with GS 10 PC, compared to 79.00% (95% CI, 61.40% to 100.0%) for those with GS 8–9.

### 3.3. Genomic Alterations and Gene Expression Analysis

**Figure 2:**
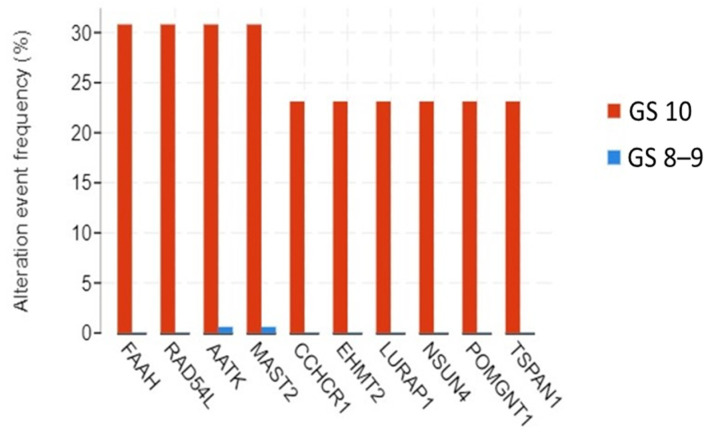
Comparison of genomic alterations between Gleason score (GS) 10 and GS 8–9. The genes shown are those with significant differences, defined by *p*-values < 0.05.

Genomic alteration analysis was conducted to evaluate the alteration event frequencies of genes. Figure 2 shows a bar plot of alteration event frequency (in each of GS 8–9 and GS 10 cohorts) for ten genes identified to be more frequently altered in GS 10 patients. Specifically, the alteration event frequencies of *FAAH*, *RAD54L*, *AATK*, *MAST2*, *CCHCR1*, *EHMT2*, *LURAP1*, *NSUN4*, *POMGNT1*, and *TSPAN1* were significantly higher in tumor tissues of patients with GS 10 compared to GS 8–9 (*p* < 0.05, Figure 2). We refer to these 10 genes as DAGs in the remainder of the study.

Gene expression analysis from database RNA sequencing data was also conducted to identify genes that varied in expression between the two Gleason score cohorts. A total of 932 DEGs were detected in gene expression analysis, including 583 genes over-expressed in GS 10 and 349 genes over-expressed in GS 8–9.

### 3.4. Protein–Protein Interaction Network and Hub Genes

**Figure 3:**
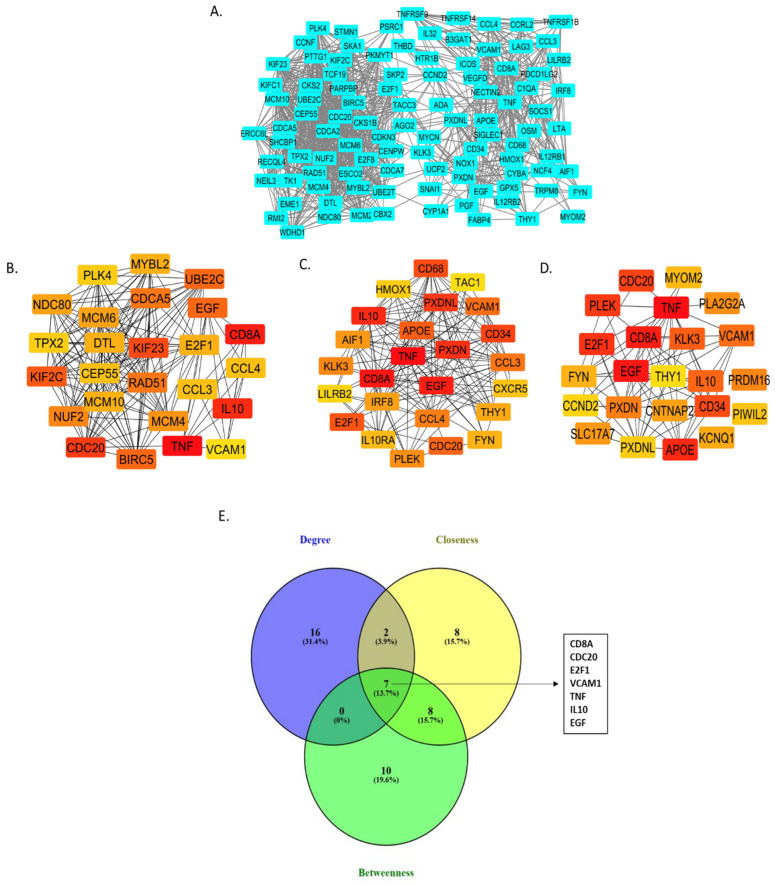
Protein–protein interaction network analysis of differentially expressed genes in patients with GS 10. (**A**) The module with the highest molecular complex detection score (17.674). The hub genes were identified using degree (**B**), betweenness (**C**), and closeness (**D**) algorithms in the Cytohubba plug-in of the Cytoscape software v3.10.1. The nodes represent proteins, and the edges represent the predicted functional associations. Red color represents the highest degree of connectivity for a protein, while yellow indicates the lowest. (**E**) The Venn diagram illustrates the overalapping hub genes identified through the MCODE and Cytohubba analyses using the degree, betweenness, and closeness algorithms.

Figure 3 summarizes the PPI network analysis conducted to identify hub genes. The constructed PPI network for GS 10 patients contained 872 protein nodes and 2905 interactions, with enrichment analysis yielding a highly significant *p*-value of 1.0 × 10^−16^. The highest-scoring MCODE module (17.674) was selected for detailed visualization (Figure 3A) and consists of 185 nodes and 1626 edges, representing protein interactions in patients with GS 10. The top 25 hub genes were identified using degree, betweenness, and closeness algorithms in the Cytohubba plugin, with results shown in Figure 3B–D, respectively. Additionally, a Venn diagram intersecting hub genes from the degree, betweenness, and closeness algorithms identified seven overlapping genes (*CD8A*, *CDC20*, *E2F1*, *EGF*, *IL10*, *TNF*, *VCAM1*) in GS 10 patients (Figure 3E). These overlapping hub genes illustrate the common intersecting genes for GS 10 and we focused on these genes in particular for further analysis of gene expression and prognostic value.

**Figure 4:**
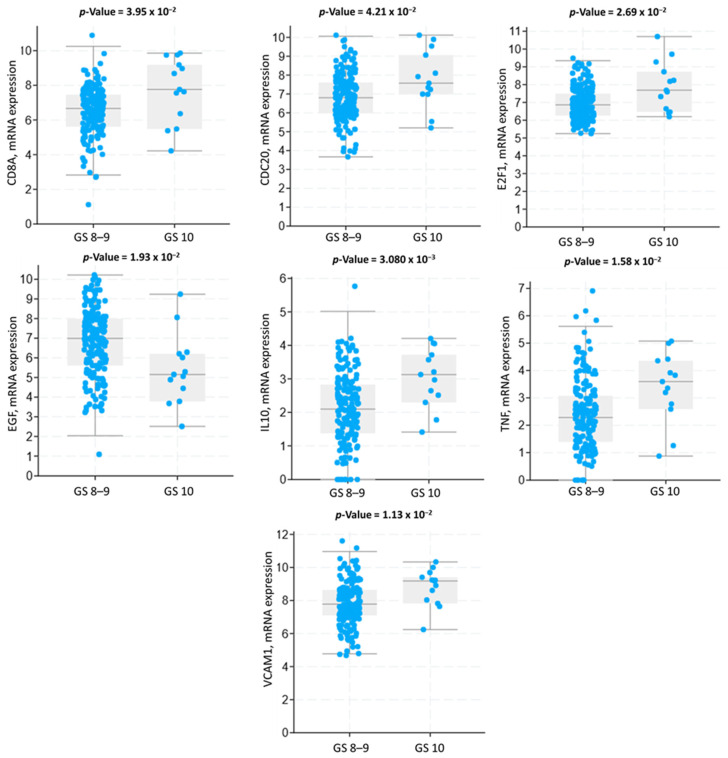
The mRNA expression level of the overlapping hub genes. Abbreviations: GS, Gleason score.

Analyzing mRNA expression data allowed us to identify how the overlapping hub genes varied in expression across GS 8–9 vs. 10 patients. Figure 4 shows how the overlapping hub genes vary in mRNA expression between the two GS cohorts. Six overlapping hub genes (*CD8A*, *CDC20*, *E2F1*, *EGF*, *IL10*, *TNF*, *VCAM1*) had significantly increased expression in GS 10 patient tumor tissue compared to those with GS 8–9, while one overlapping hub gene (*EGF*) had significantly lower expression in the tumor tissues of patients with GS 10 compared to those with GS 8–9 (Figure 4).

### 3.5. The Prognostic Value of the Altered and Hub Genes

**Figure 5:**
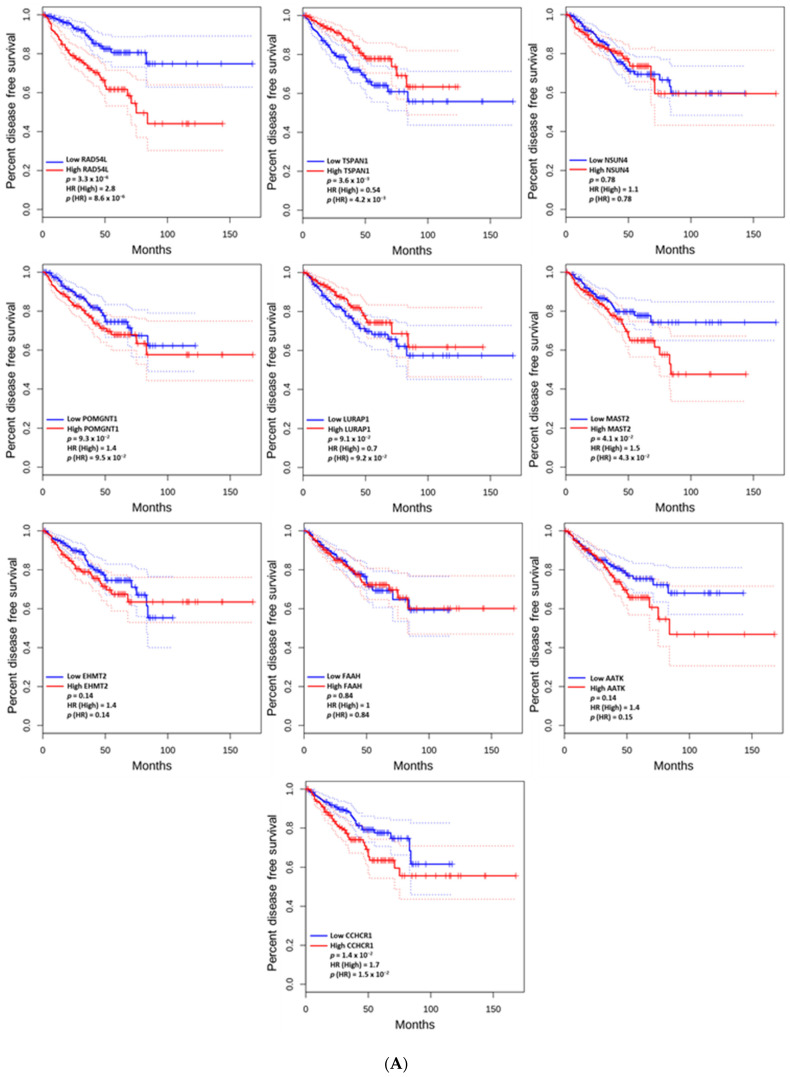

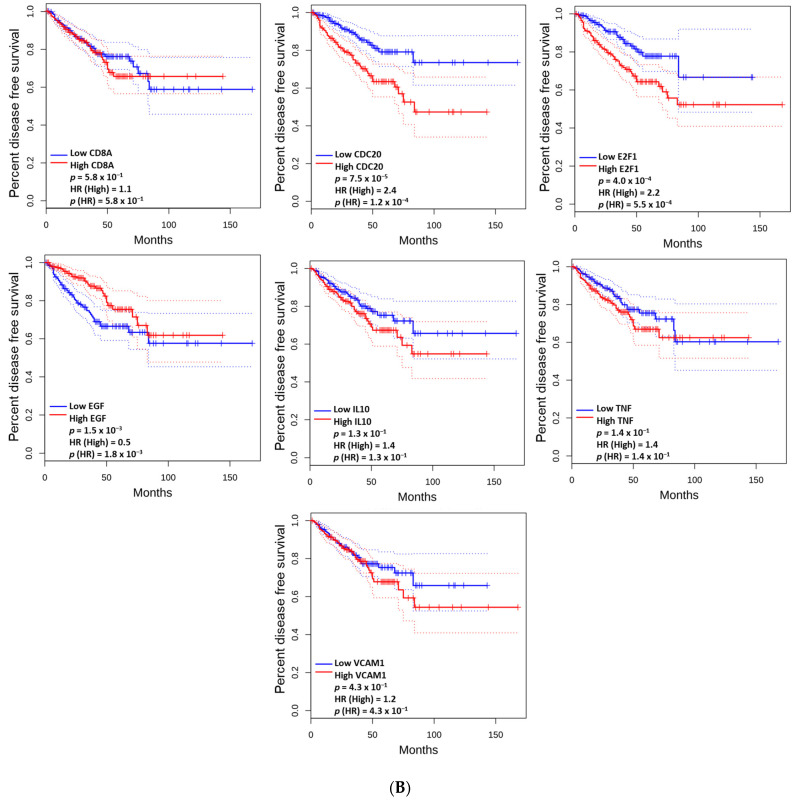
The disease-free survival (DFS) based on the DAGs (**A**) and the hub genes (**B**). The DFS curve is represented by the solid line, bound by dotted lines representing the 95% confidence interval. Blue lines represent cohorts with lower expression of a specific gene of interest, and red lines represent cohorts with higher expression of that gene. The hazard ratio (HR) measures the relative risk of an event between the high- and low-expression groups, while p(HR) assesses its statistical significance, with values below 0.05 indicating a meaningful difference. A log-rank test *p*-value < 0.05 was considered statistically significant.

Figure 5A plots the association between mRNA expression levels of the previously identified DAGs (the ten genes identified with significantly higher alteration rates in GS 10 patients compared to GS 8–9) and patient DFS. Among the DAGs, high mRNA expression levels of *CCHCR1* (*p* = 1.4 × 10^−2^), *MAST2* (*p* = 4.1 × 10^−2^), and *RAD54L* (*p* = 3.3 × 10^−6^) were associated with a shorter DFS whereas *TSPAN1* (*p* = 3.6 × 10^−3^) was associated with longer DFS (Figure 5A).

Figure 5B performs a similar analysis but instead compares mRNA expression of the overlapping hub genes with DFS. Among the overlapping hub genes, high mRNA expression levels of *CDC20* (*p* = 7.5 × 10^−5^), and *E2F1* (*p* = 4.0 × 10^−4^) were associated with a shorter DFS, whereas *EGF* (*p* = 1.5 × 10^−3^) was associated with a longer DFS (Figure 5B).

### 3.6. Functional Enrichment Analysis

**Figure 6:**
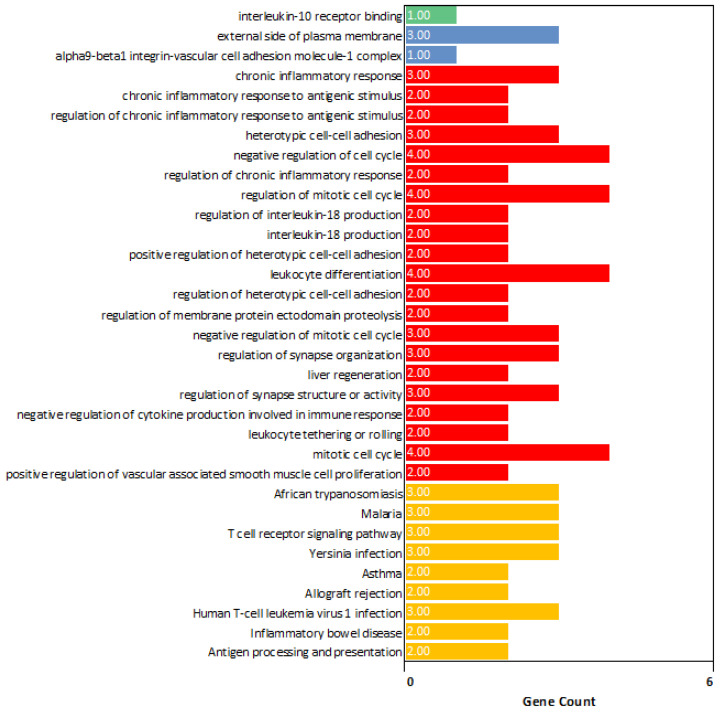
Gene ontology (GO) and pathway enrichment analysis of differentially expressed hub genes. The x-axis represents the number of enriched differentially expressed hub genes, and the y-axis shows the enriched GO and KEGG pathway terms. Green bars represent molecular function, blue bars represent cellular components, red bars represent biological processes, and yellow bars represent KEGG pathways.

Functional enrichment analysis (Figure 6) highlighted key biological processes, molecular functions, and cellular components associated with prostate cancer pathology in GS 10 patients. GO biological process analysis revealed alterations in chronic inflammatory response (GO:0002544), chronic inflammatory response to antigenic stimulus (GO:0002439), and interleukin-18 production (GO:0032621), suggesting their role in the unique biology of GS 10 PC. GO molecular function analysis identified important activities, including interleukin-10 receptor binding (GO:0005141). GO cellular component analysis highlighted structures such as the external side of the plasma membrane (GO:0009897) and the alpha9-beta1 integrin-vascular cell adhesion molecule-1 complex (GO:0071065). Additionally, KEGG pathway analysis showed enrichment in the T cell receptor signaling pathway (KEGG:04660) and antigen processing and presentation (KEGG:04612), emphasizing immune-related mechanisms in GS 10 patients.

## 4. Discussion

In this study, we found that in patients with PC who underwent RP, the presence of GS 10 was associated with distinct genomic alterations and lower time to BCR after adjusting for known prognostic factors. The clinical significance of these findings is their potential to guide the development of novel therapeutic targets, prognostic biomarkers, and more personalized treatment approaches for patients with GS 10 PC.

Several points require further discussion. First, consistent with previous reports, our findings demonstrate that patients with GS 10 PC had a significantly shorter time to BCR than those with lower GS, even after controlling for key prognostic factors such as age, preoperative PSA, and pathologic stage. This highlights the aggressive nature of GS 10 disease, which is likely driven in part by a high burden of micrometastatic spread at initial presentation. Indeed, GS 10 tumors often present with more advanced pathological features—such as extracapsular extension and seminal vesicle invasion [18]—which indicates their propensity for broader disease dissemination. Current treatment strategies for high-risk PC generally comprise either RP with pelvic lymph node dissection or radiation therapy combined with androgen deprivation therapy (ADT), with abiraterone added in selected very-high-risk cases. For patients experiencing PSA persistence or recurrence following surgery, salvage radiotherapy—initiated at a PSA threshold of 0.1 ng/mL—with or without ADT has become standard, reflecting evidence from multiple trials showing no clear benefit for adjuvant over salvage RT in most patients [19,20,21,22]. However, recent data suggest that a subset of men characterized by very high-risk features (e.g., GS 8–10 and pT3–4 disease) may indeed derive an all-cause mortality benefit from adjuvant treatment [23]. These findings support the need for personalizing therapeutic strategies following RP in patients who are at high risk for disease recurrence.

Second, the genomic analysis identified distinct alterations in several key genes associated with GS 10 prostate cancer, with implications for disease progression and prognosis. Notably, *RAD54L*, *FAAH*, *AATK*, and *MAST2* were among the genes that were altered more in GS 10 patients compared to GS 8–9 patients. *RAD54L*, known for its role as an oncogene, is associated with tumor progression and poor prognosis in multiple cancers [24], and has potential as a therapeutic target. Similarly, *FAAH* functions as a metastasis suppressor, and its high expression correlates with worse outcomes in luminal breast cancer [25], suggesting its inhibition could enhance anti-tumor effects. *AATK*, a tumor suppressor gene frequently hypermethylated in cancer, has shown promise for therapeutic reactivation to improve outcomes [26]. Overexpression of *MAST2*, an oncogene involved in tumor proliferation and survival, is linked to poor prognosis in liver cancer and represents another potential treatment target [27]. Additionally, high mRNA expression levels of genes such as *RAD54L* (*p* = 3.3 × 10^−6^), *MAST2* (*p* = 4.1 × 10^−2^), and *CCHCR1* (*p* = 1.4 × 10^−2^) were associated with shorter DFS, further highlighting their role in tumor aggressiveness. Conversely, high expression of *TSPAN1* (*p* = 3.6 × 10^−3^) was linked to longer DFS, suggesting its potential as a protective biomarker. These findings emphasize the unique molecular characteristics of GS 10 tumors and highlight opportunities for personalized therapeutic strategies targeting these genes.

Third, building upon these findings, our analysis also revealed that several hub genes (*CD8A*, *CDC20*, *E2F1*, *IL10*, *TNF*, and *VCAM1*) were significantly overexpressed in GS 10 tumors compared to patients with GS 8–9, whereas one overlapping hub gene (*EGF*) was significantly lower in the tumor tissues of patients with GS 10 compared to those with GS 8–9. These genes play diverse and critical roles in cancer biology, immune responses, and cell signaling pathways, reflecting the complex mechanisms driving aggressive tumor behavior in patients with GS 10 PC. For instance, *CD8A* is essential for cytotoxic T-cell-mediated anti-tumor immunity, and its overexpression may indicate alterations in immune surveillance [28,29]. Specifically, *CD8A* could be overexpressed in tumor samples due to increased T-cell activity; its expression could imply that the immune system is mounting a stronger response to the aggressive GS 10 cancer. Improving cytotoxic T-cell immunity against cancer is a key goal of cancer immunotherapy, as such GS 10 cancer could be an interesting candidate for future immunotherapeutic study. *CDC20*, a cell cycle regulator promoting mitosis, is frequently overexpressed in cancers and associated with poor prognosis [30]. Overexpression of *CDC20* promoting cancer cell division could be a driving force behind the aggressiveness seen in GS 10 patients [31]. This has been supported by a study that found that *CDC20* is important to the function of prostate cancer stem-like cells—cells that are involved in cancer spreading and relapse. Similarly, *E2F1* functions as a mediator of cell proliferation, migration, and survival pathways, highlighting its role in enabling tumor growth and metastatic potential [32,33,34]. Another study also found *E2F1* overexpression in PC patients with GS of at least 8 compared to patients with lower GS [35]. It is interesting that our study finds that E2F1 protein is further overexpressed in GS 10 patients compared to GS 8–9, implying that *E2F1* expression could delineate a gradient of PC aggressiveness. *EGF*, a central player in the *EGF/EGFR* signaling pathway, regulates cell growth and differentiation, exemplifying the intricate signaling mechanisms active in these tumors [36]. *EGF*’s role in regulating cell growth and differentiation highlights the complexity of signaling pathways that influence tumor behavior [37]. *IL-10*, an anti-inflammatory cytokine, may play a complex role in cancer progression. For instance, studies have shown that *IL-10* can promote immune evasion in advanced cancers, potentially contributing to tumor aggressiveness [38]. Additionally, elevated *IL-10* levels have been associated with poor prognosis in high-grade prostate cancer, which may contribute to the aggressive nature of GS 10 disease [39]. It is important to note that while our results align with previous research on *IL-10* and its role in immune evasion, its specific impact on the progression of GS 10 prostate cancer requires further investigation. *VCAM1* plays important roles in cell adhesion and intracellular signaling, processes critical for tumor progression and metastasis [40,41]. Finally, *TNF*, a multifunctional cytokine, shows the inflammatory component of cancer progression, with its ability to promote inflammation, cell survival, or apoptosis [42,43]. Collectively, these findings further emphasize the unique molecular characteristics of GS 10 tumors, even distinct from the rest of “high-risk” diseases (GS 8–9). Continued exploration of these hub genes could lead to the discovery of novel biomarkers and treatment approaches for this aggressive PC subset. These findings also suggest that future treatment paradigms may need to be individualized specifically for GS 10 disease to optimize the outcomes for these patients.

Finally, GO analysis indicated that GS 10 patients had alterations in many immune-related processes, such as chronic inflammatory response, chronic inflammatory response to antigenic stimulus, and interleukin-18 production. These findings need clinical and laboratory analysis but imply that the immunological implications of genomic differences in GS 10 prostate cancer merit further study.

Despite offering important insights into the molecular profile of GS 10 PC, this study has several limitations that merit attention. First, this is a hypothesis-generating retrospective study that needs validation in a larger, independent cohort. Specifically, our study involved low numbers of GS 10 patients due to the rarity of GS 10 PC; a study that includes more GS 10 patients is ideal but practically difficult. Second, the TCGA database does not capture detailed post-RP longitudinal PSA data or radiology findings, limiting our ability to perform nuanced assessments of treatment outcomes. Third, the lack of detailed information regarding adjuvant therapies may have influenced the observed time to BCR outcomes, highlighting the need for comprehensive treatment data to accurately interpret the disease trajectory. Finally, functional validation studies, including in vitro co-culture models or in vivo experiments, would provide more definitive evidence linking the identified molecular alterations to the aggressive phenotype observed in GS 10 PC. Another future direction would be to conduct protein network and gene expression analyses specifically focusing on established pathways in prostate cancer biology, again comparing GS 10 with other forms of prostate cancer. Such further work could integrate pathway-specific analyses related to androgen signaling, tumor suppressors, and DNA damage response to complement our findings and assess their therapeutic implications.

## 5. Conclusions

This study highlights the aggressive nature of GS 10 PC and its distinct genomic landscape, which collectively contribute to poor clinical outcomes. These findings indicate the need for further research exploring personalized treatment intensification strategies and biomarker-driven approaches to improve outcomes for this very high-risk patient population.

## Figures and Tables

**Table 1:** Comparison of the distribution of clinical and treatment factors stratified by Gleason score.

	Gleason Score 8–9 (n = 179)	Gleason Score 10 (n = 13)	*p*
**Age (years), median (IQR)**	63 (57, 66.5)	65 (60, 68)	0.193
**Baseline PSA, ng/mL, median (IQR)**	8.9 (5.35, 14.8)	14.2 (8.2, 64.1)	0.038
**Prostatectomy tumor stage, No. (%)**			0.200
T2	27 (15%)	0 (0%)	
T3a or higher	152 (85%)	13 (100%)	
**Prostatectomy margin status, No. (%)**			<0.001
Negative	90 (50%)	0 (0%)	
Positive	89 (50%)	13 (100%)	
**Prostatectomy nodal status, No. (%)**			0.400
Negative (N0)	123 (69%)	7 (54%)	
Positive (N1)	56 (31%)	6 (46%)	
* Follow-up (month), median (IQR)	37.87 (16.53, 64.37)	43.40 (19.30, 70.23)	0.002

Abbreviations: PSA, prostate-specific antigen. * Reverse KM method is used to estimate time of median follow-up.

**Table 2:** Covariate-adjusted hazard ratios for time to biochemical recurrence.

Covariates	Univariable			Multivariable		
	HR	95% CI	*p*	AHR	95% CI	*p*
**Age (years)**	1.004	0.966–1.044	0.825	1.003	0.996–0.965	0.860
**Baseline PSA, ng/mL**						
<4	0.939	0.407–2.170	0.884	1.058	0.443–2.528	0.898
4–10	Reference	Reference		Reference	Reference	
>10	1.122	0.890–1.901	0.667	0.934	0.532–1.638	0.813
**Prostatectomy tumor stage**						
T2	Reference	Reference		Reference	Reference	
T3a or higher	2.535	0.919–6.991	0.072	2.213	0.793–6.175	0.129
**Prostatectomy margin status**						
Negative	Reference	Reference		Reference	Reference	
Positive	1.489	0.894–2.483	0.126	1.206	0.658–2.217	0.545
**Prostatectomy nodal status**						
Negative (N0)	Reference	Reference		Reference	Reference	
Positive (N1)	1.227	0.724–2.080	0.446	1.153	0.657–2.023	0.619
**Prostatectomy Gleason score**						
8–9	Reference	Reference		Reference	Reference	
10	3.148	1.485–6.676	0.002	2.669	1.183–6.023	0.018

Abbreviations: HR, hazard ratio; CI, confidence interval; AHR, adjusted hazard ratio; PSA, prostate-specific antigen.

## Data Availability

The original contributions presented in the study are included in the article. Further inquiries can be directed to the corresponding author.
